# Psychometric analysis of the Generalized Anxiety Disorder scale (GAD-7) in primary care using modern item response theory

**DOI:** 10.1371/journal.pone.0182162

**Published:** 2017-08-03

**Authors:** Pascal Jordan, Meike C. Shedden-Mora, Bernd Löwe

**Affiliations:** 1 Department of Psychosomatic Medicine and Psychotherapy, University Medical Center Hamburg-Eppendorf and Schön Klinik Hamburg Eilbek, Hamburg, Germany; 2 Department of Psychological Methods, Faculty of Psychology and Movement Sciences. University of Hamburg, Hamburg, Germany; TNO, NETHERLANDS

## Abstract

**Objective:**

The Generalized Anxiety Disorder scale (GAD-7) is one of the most frequently used diagnostic self-report scales for screening, diagnosis and severity assessment of anxiety disorder. Its psychometric properties from the view of the Item Response Theory paradigm have rarely been investigated. We aimed to close this gap by analyzing the GAD-7 within a large sample of primary care patients with respect to its psychometric properties and its implications for scoring using Item Response Theory.

**Methods:**

Robust, nonparametric statistics were used to check unidimensionality of the GAD-7. A graded response model was fitted using a Bayesian approach. The model fit was evaluated using posterior predictive p-values, item information functions were derived and optimal predictions of anxiety were calculated.

**Results:**

The sample included N = 3404 primary care patients (60% female; mean age, 52,2; standard deviation 19.2) The analysis indicated no deviations of the GAD-7 scale from unidimensionality and a decent fit of a graded response model. The commonly suggested ultra-brief measure consisting of the first two items, the GAD-2, was supported by item information analysis. The first four items discriminated better than the last three items with respect to latent anxiety.

**Conclusion:**

The information provided by the first four items should be weighted more heavily. Moreover, estimates corresponding to low to moderate levels of anxiety show greater variability. The psychometric validity of the GAD-2 was supported by our analysis.

## 1. Introduction

With a point prevalence of about 8% Generalized Anxiety Disorder (GAD) is the most frequent anxiety disorder in primary care [1]. GAD is statistically associated with age and gender [2] and shows high comorbidity with depression and somatization [1, 3]. Patients affected with GAD are impaired in their daily life and work life. The latter results in severe numbers of disability days [4] and correspondingly increased economic costs. From the perspective of the primary care physician it is important to identify GAD in a reliable and yet economic way in order to initiate a proper treatment–e.g. psychotherapy—and in order to decrease the economic cost resulting from the usage of additional health services due to somatic symptoms. In the absence of brief and validated measures of anxiety [5], the GAD-7 [4] was developed to provide a brief self-report measure to identify generalized anxiety in primary care. Relevantly, primary care physicians often struggle to recognize signs of psychological distress, particularly anxiety, despite being the first, if not the only point of contact with the healthcare system [6]. Therefore, an easily applicable and face valid measure for GAD has the potential to improve recognition of GAD and address a pressing public health need.

The GAD-7 has been validated within a large sample of patients in a primary care setting [4] and also within a large general population sample in Germany [2]. Based on the results of applying the paradigm of classical test theory (CTT)—as well as the corresponding confirmatory factor analysis (CFA) methods—a one-factorial structure has been claimed and established, a high (and in addition subpopulation invariant) internal consistency (α = 0.89) has been reported and reference scores based on norms for the general population have been provided [2].

In this study we examined the GAD-7 scale with the help of the item response theory (IRT) paradigm–a paradigm which is more suited to the ordered categorical response format of the items (in the same way as logistic regression or ordinal regression is more appropriate than ordinary linear regression when the outcome is not measured on an interval scale) and which at the same time provides opportunities to establish and link scores obtained from applying shortened versions of the GAD-7. The core notion underlying IRT is the idea that a certain characteristic of individuals (i.e., the latent ability) determines the probability of responding to an item (i.e., the manifest indicator) in a certain manner. Based on the responses to the items, the underlying mathematical model allows for the derivation of a person’s latent “ability” (e.g., anxiety severity). As clear advantages in contrast to classical test theory based models, IRT models are suited to the categorical response format without having to pose the far-fetched assumption of a metric measurement scale. In addition, IRT models do not confound item difficulty with item discrimination (i.e. in CTT items are often excluded from a scale due to extreme item difficulty). They also lead to more realistic confidence intervals for a person’s latent ability–that is, a CTT model implies an unrealistic uniformity of intervals, wherein the precision of measurement is independent of the person’s true score. In case of the GAD-7 this has to be emphasized: The practitioner should not treat different scores with the same type of confidence. The present IRT analysis aims to reveal which levels of anxiety can be inferred with reasonable precision and which levels are only assessable in a vague form. Clearly, applying ordinary CTT is rather misleading in this regard, as different levels would be treated equally in terms of confidence levels.

However, to date IRT analysis in clinical psychological diagnostics are relatively rare. With a few exceptions [7, 8] the commonly applied approach to the analysis of the GAD scale is based on classical test theory (CTT). Nevertheless, from a methodological point of view, the IRT approach is in general favored over the CTT approach. This striking discrepancy between the practical application (wherein CTT based analysis outweigh IRT based analysis by far) and the theoretical ideal has led Borsboom to conclude in one of the leading psychometric journals: “*As such, contemporary test analysis bears an uncanny resemblance to the psychometric state of the art as it existed in 1950s” ([9], p.425).*

Beside the mentioned methodological advantages of IRT, clinical benefits include the interpretability of the scale even if several items are missing. Moreover, while a symptom score within the CTT framework only provides information on symptom severity, a score within the IRT framework reveals probabilistic information about item endorsement (e.g, being on the 90^th^ percentile on the GAD-7 scale relates to a # % probability of not being able to stop or control worrying) [10].

The aim of this study was to conduct a state of the art IRT analysis which allows for the establishment of a common item metric–thus enabling the usage of shortened versions of the GAD-7 without losing comparability of test scores. The main scope of the analysis is to improve the application of the scale by deriving a superior method of weighting the item information and by also providing more realistic methods to assess the precision and confidence level of a score level. In addition, brief versions of the GAD-7 are evaluated. Thereby, an IRT analysis might simplify the diagnostic process and improve accuracy in clinical practice.

We would also like to stress the importance of any valid and economically applicable method to detect GAD in the primary care setting. In many cases the primary care physician is the first (and only) contact of patients with GAD. Thus, a lot of cases will remain undetected (with potentially severe consequences for the patient but also for societal costs as GAD-patients are frequent health care users). It is therefore a fundamental need to supply primary care physicians with an instrument which allows for a more precise detection and which at the same time fits into the daily routine that is frequently characterized by time pressure.

The rest of the paper is structured as follows: We first provide data on the characteristics of our sample (section 2), then present CTT-derived statistics and a brief CFA analysis (section 3.1). This will not only serve as a level of comparison for the IRT approach but will also allow the practitioner to judge the functioning of the scale in our sample with respect to different samples for which a CTT analysis has already been conducted. The main part of the paper–located in section 3.2 –is concerned with assessing the unidimensionality of the GAD-7. We first describe and apply a highly robust method to assess unidimensionality. We then head towards the establishment of a common metric by fitting a graded response model via the Bayesian paradigm which also allows for the proper handling of missing data.

As a result of this, item characteristics are obtained, lack of fit is analyzed and methods for an adequate inference of GAD based on shortened versions of the GAD-7 are commented on. It is also highlighted (and a precise formulation is provided in the appendix) that the items should be weighted differently, when inferring the level of GAD (note that here the IRT approach is crucial in order to derive a reasonable method of weighting the items as the CTT approach would intermingle the concept of item difficulty and item discrimination–see the preceding remarks above.). The concluding remarks highlight potential lines of further research in order to improve the model fit and also provide a link to other anxiety measures via the study of Schalet et al. [8].

## 2. Methods

### 2.1. Design and sample

The sample consists of 3404 primary care patients who were assessed in the context of the Sofu-Net study which aimed to establish and evaluate a health care network for the treatment of somatoform disorders (following the pre/post-treatment evaluation study described in [11]; Ethics approval was obtained from the Medical Chamber Hamburg.). The patients were recruited in 33 different primary care practices in Hamburg. More specifically, in each practice patients (after providing oral informed consent) provided answers with respect to a) demographic characteristics; b) characteristics referring to the usage of health care services and c) a battery of items from the Patent Health Questionnaire (PHQ)–consisting of the PHQ-15 scale (measuring somatic symptom burdens), the PHQ-9 scale (measuring depression) and the GAD-7 scale. The practices were approached on two to four consecutive days. Severe somatic or psychiatric diseases, acute suicidality, severe cognitive disabilities, being younger than 18 years old, having impaired vision and insufficient German language skills were the key variables which lead to the exclusion of the patient (for further information see [12]).

### 2.2. Instruments

Although the study included the whole Patient Health Questionnaire (PHQ), our analysis is focused on the GAD-7. The latter was developed in a primary care setting to fill in the gap for having a brief measure for the assessment of GAD. The GAD-7 represents an anxiety measure based on seven items which are scored from zero to three. The whole scale score can range from 0 to 21 and cut-off scores for mild, moderate and severe anxiety symptoms are 5, 10 and 15 respectively [13]. At the cut-off score of 10 both sensitivity as well as specificity exceed .0.8, so that the operating characteristic of the scale–based on using a structured psychiatric interview as the criterion—is satisfactory [13]. Internal consistency of the GAD-7 was estimated at 0.92 and convergent validity was established by means of correlations with two other anxiety measures [4]. Importantly for our purposes, the development of the GAD was not based on a latent variable model but motivated by reasons relying on the classical test theory concept of item-total-correlation–namely the incorporation of the seven most discriminating items among 13 candidate items.

### 2.3. Statistical analysis

As a level of comparison we first conducted a CTT based analysis using item-total correlations and the confirmatory factor analysis framework to test the dimensionality hypothesis. The main part was focused on the IRT analysis and used elementary nonparametric statistics in order to judge the unidimensionality hypothesis without having to impose a restrictive response model (as was the case in the above CTT-based framework wherein a highly restrictive model was fitted). With respect to this goal we note that the following implications need to hold, if the underlying IRT model is indeed unidimensional [14, 15]:

1. Nonnegativity of all pairwise item correlations.
2. Nonnegativity of all conditional pairwise item correlations. (This follows from Theorem 6 in [14])
3. Monotonicity of the conditional distribution functions of the item scores given the restscore. (see Theorem 5 in [14]—this requires some additional assumption like e.g. the latent TP2 assumption.)

The above mentioned necessary requirements were checked based on suitably defined statistics (see section 3). For the establishment of a common metric of item threshold and person parameters we used the graded response model which was fitted via the Bayesian approach to handle missing data appropriately. For evaluating model fit, we apply posterior-predictive p-values which were derived by Markov Chain Monte Carlo methods. Detailed comments on the procedures are also given in the following section.

## 3. Results

The sample consists of 3404 primary care patients who were recruited within 33 different primary care practices in Hamburg. The total response rate was 73,3%. The main demographic variables–age (range 18–98) and gender–as well as the variables related to the Patient Health Questionnaire are summarized in Table 1.

**Table 1:** Sociodemographic characteristics and key variables of the patient health questionnaire.

	*mean*	*sd*	*first quartile*	*median*	*third quartile*	*n*_*eff*_
**Variable**						
gender	0.60					3391
age	52.18	19.20	35.00	51.50	69.00	3388
PHQ-9	5.20	5.06	2.00	4.00	7.00	2979
PHQ-15	7.04	4.69	3.00	6.00	10.00	3262
GAD-7	4.75	4.76	1.00	3.00	7.00	3303

*Note*. gender is coded binary with “1” indicating female. The different effective numbers of sample size (neff) result from missing data–i.e. the computation of each statistic was based on all available cases for that variable.

### 3.1. Classical test theory based analysis of the GAD-7

In Table 2 the pairwise item correlations are depicted. As can be seen, the pairwise item correlations are nonnegative (a rudimentary requirement for unidimensionality) and range between 0.45 and 0.74. Item-total correlations are also shown in Table 2. These values could be used to discern the items with respect to their discriminatory power–however, using these values can be misleading due to the categorical nature of the data. That is, the marginal distributions of the item scores restrict their pairwise correlations and their item-total correlations [16]. In particular, items whose item difficulties are located outside of the remaining bulk of items are (mis)judged as low discriminating items when using the classical test theory based measure of item-total correlation. Thus, it is useful to have an alternative measure of item discrimination which is not confounded with item difficulty. The scalability coefficients according to Loevinger share this property. They represent a nonparametric measure of an items discrimination which is corrected for discrepancies in marginal distributions (for details we refer the reader to [17] and [16]). For the data set at hand, it can however be gleaned from the corresponding column of Table 2 that the ordering of the items based on item-total-correlation is almost identical with the ordering based on the scalability coefficients–the only minor difference is the interchange between the third and the fourth item.

**Table 2:** Item characteristics of the GAD-7.

Item	*M*	*SD*	R	Hi	1	2	3	4	5	6
1	0.80	0.92	0.73	0.62						
2	0.59	0.85	0.78	0.67	.66**					
3	0.76	0.91	0.75	0.64	.62**	.74**				
4	0.90	0.95	0.75	0.66	.62**	.63**	.63**			
5	0.46	0.78	0.60	0.55	.47**	.47**	.45**	.56**		
6	0.74	0.82	0.62	0.55	.47**	.50**	.50**	.54**	.49**	
7	0.51	0.80	0.69	0.61	.59**	.62**	.59**	.54**	.45**	.48**

Means (M), standard deviations (SD), item-restscore correlations (R), scalability coefficients (Hi) and pairwise correlations of the GAD-7-items.

*Note*.

* indicates p < .05;

** indicates p < .01. M and SD are used to represent mean and standard deviation, respectively. R and Hi are used to represent item discrimination.

As a CTT-based measure of reliability we followed the approach outlined in Raykov [18] and computed a weighted scoring of the items (after fitting of an ordinary linear one-factor model) such that the resulting composite score has maximal reliability among all possible scoring methods. The resulting weights were 2.12, 3.42, 2.76, 2.12, 1.24, 1.32, 1.94 and the estimated reliability of this score was 0.91 with 95%-Bootstrap-CI: (0.90, 0.92). Note that we refrain from computing Cronbach’s alpha due to various drawbacks of this coefficient [19].

We fitted a CFA model with ordered categorical data—hence using the polychoric correlation matrix—using the R package lavaan [20]. To ensure identifiability, the loading of the first item was fixed at the value 1. The estimated parameters can be obtained from the authors. The fit measures with method “DWLS” were: RMSEA = 063 (90%-CI: 0.055–0.071), CFI = 0.997, TLI = 0.996, SRMR = 0.037. The robust versions of these statistics were somewhat lower but still indicating reasonable model fit: RMSEA = 0.096 (CI: 0.089–0.104), CFI = 0.988, TLI = 0.982, SRMR = 0.037. Following conventional guidelines [21], the fit of the CFA model can be judged as reasonably good–supporting the underlying unidimensionality hypothesis.

### 3.2. Item response theory based analysis of the GAD-7

We first checked unidimensionality via the three general criteria which were outlined in the statistical analysis part of section 2. As was already noted, 1) holds for the items of the GAD7-scale. With respect to 2) we examined all pairwise correlations of two items i and j conditional on fixing the performance of another reference item l to a prescribed value X_l_ = c. Iterating this procedure for all possible values of l and c we arrive at a summary measure with respect to the violation of 2)–namely the number of negative conditional item covariances. For the items of the GAD-7 not a single violation with respect to the sign was counted. Therefore, the descriptive statistic underlying 2) does also support the unidimensionality hypothesis. Turning to 3) and denoting with X_j_ the item score on the j-th item, we examined the monotonicity of the following function (which ought to be monotone in k):

$$f_{i}\left(k\right)≔\;P\left({\text{X}}_{i}\geq c|S_{-i}=k\right)\;with\;S_{-i}=\underset{j\neq i}{\sum }X_{j}$$

The corresponding calculations were done in the R package mokken [22] using the default settings. Not a single violation can be reported for the items of the GAD-7.

- Hence, the examination of item association is in line with the association structure which is to be expected from a unidimensional latent variable model. This provides evidence for the unidimensionality hypothesis on firmer grounds than an examination of the CFA output, due to a) the lack of restrictive parametric assumptions, b) the absence of computational obstacles, and c) the independence of the results of the true distribution of the latent variable (in our case “anxiety”) and of potential nonlinearities.

The unidimensionality check supports the usage of the sum score for the ordering of subjects with respect to their latent anxiety. However, it does not discern the possibly different quality of the items nor does it provide a method to construct shortened versions of the GAD-. In the following we therefore turn to the fitting of (restrictive) parametric item response models which enable the delineation of item and person parameters on a common metric.

In order to deal with missing data appropriately (assuming MAR, see [23]) we employ a fully Bayesian analysis of the data via the software WinBUGS [24]. More precisely, we estimate the model parameters of a graded response model via draws from their posterior distribution based on the complete data set (the missing data are implicitly handled by augmenting the parameters and sampling from the joint posterior). The corresponding BUGS Code can be found in [25]. We used five chains, 6000 iterations, a burn- in of 2000 and a total amount of retained samples of 1000. Convergence was assessed via graphical methods (summaries are available on request). Table 3 contains the estimated parameters.

**Table 3:** Parameter estimates for a graded response model (with logistic errors).

	*λ*	*τ*_*1*_	*τ*_*2*_	*τ*_*3*_
Feeling nervous anxiety	2.64	-0.46	2.83	4.27
Not able to stop worrying	3.55	0.74	4.24	6.37
Worrying too much	3.10	-0.20	3.20	5.07
Trouble relaxing	2.74	-0.78	2.30	4.25
Hard to sit still	1.69	1.04	2.94	4.30
Becoming easily annoyed	1.69	-0.36	2.44	4.06
Something might happen	2.32	0.93	3.53	4.86

*Note*. λ represents the item discrimination. The τ-parameters are item thresholds which are used in the model to categorize the latent continuous measurements into the observable ordered categorical item scores (see e.g. [25]). Note that our model differs slightly from the conventional graded response model as the error distribution is assumed to be of logistic rather than normal shape–see [25].

In addition, to gain insight into ways of improving the model, we computed posterior predictive p-values according to the method proposed by Sinharay [26]. That is, model parameters drawn from the posterior distribution are used to simulate new data sets (of the same size as the original data set). For each simulated data set, a test statistic is computed–we use the value of the pairwise item correlations as a test statistic (for justification see [27]). The distributions (one for each item pair) of the resulting values of the statistic are then used to generate “p-values” for the data set at hand. Although these posterior predictive p-values are not p-values in the strict sense, they are a common tool (see ch. 6 of [28]) to infer model fit in the Bayesian framework [29].

In addition, simulation results [27] suggest that treating them as actual p-values does not necessarily result in increased type 1 errors, so that they possess a surplus value besides their Bayesian justification.

The pattern of the p-values (Table 4) clearly shows the direction of model misfit, as the p-values corresponding to adjacent item pairs are significantly low. Thus, the present data analysis reveals two main points:

**Table 4:** Posterior predictive p-values for the pairwise item correlations of the GAD-7.

Variable	1	2	3	4	5	6
Feeling nervous anxiety						
Not able to stop worrying	.088					
Worrying too much	.872	.000				
Trouble relaxing	.504	.552	.954			
Hard to sit still	.362	.244	.126	.000		
Becoming easily annoyed	.716	.682	.774	.006	.000	
Something might happen	.004	.188	.188	.638	.220	.034

*Note*. An entry is computed as 2min(F(tobs),1-F-(tobs)), wherein F(tobs) refers to the empirical distribution function (evaluated at the observed item correlation tobs) of the pairwise item correlations generated from simulated data sets adhering to the graded response model with item parameters drawn from the posterior distribution [26,27].

1. The GAD-7 satisfies several requirements for an unidimensional IRT model.
2. A possible way of improving model fit–beyond the model fit provided by a graded response model—would rely on the incorporation of an additional parameter representing local dependencies between adjacent items.

Although the graded response model fails in a strict sense (note however that even minor model discrepancies would be highlighted by the p-values given the large sample size), it provides a reasonable first approximation to the true model in view of the criteria cited in Schalet et al. [8].

**Fig 1:**
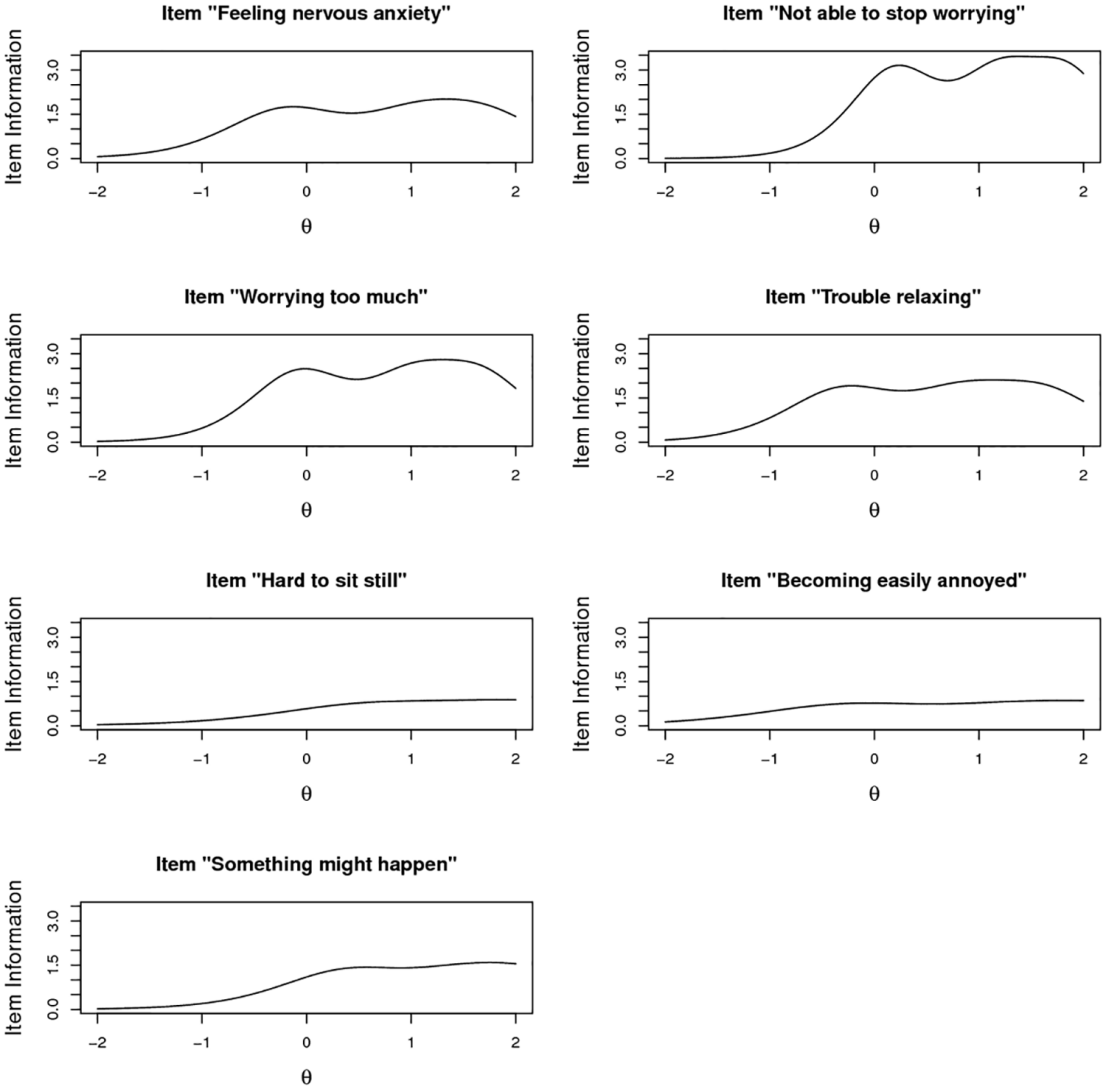
Plots of the item information functions.

We also conducted a check whether differential item functioning w.r.t. gender is present. If the latter were present then two patients of opposite sex with the same level of anxiety would have differing probability to obtain a specific score on the item. In that case, the test would be biased. In order to test for this effect, we used the lordif R-package [30]—the detailed output of the analysis is available on request) and evaluated the relative contribution of the variable gender in a logistic regression equation which also contains the anxiety variable as a predictor for the item score. The relative improvements were tiny (Nagelkerke's R^2<0.009), so DIF is negligible.We now discuss the practical implications of the above analysis: Firstly, based on the estimated model parameters—see Table 3 - (approximate) inference of a patients anxiety can be accomplished via the method described in the appendix. In general, this type of inference is a substantial improvement on the commonly applied sumscore scoring. Secondly, in developing shortened versions of the GAD-7, the item information functions can be used to judge competing choices. [The item information function for the j-th item is given by the following expression (see also [31])

$$\sum_{k}P_{jk}(\theta )[-\frac{\partial^{2}}{\partial \theta^{2}}\text{ln}\left(P_{jk}\left(\theta \right)\right)],$$

wherein *P*_*jk*_ denotes the probability of scoring in category k on the j-th item. The item information functions for the items of the GAD-7 are depicted in Fig 1.]

#### Evaluation of brief versions of the GAD-7

**Fig 2:**
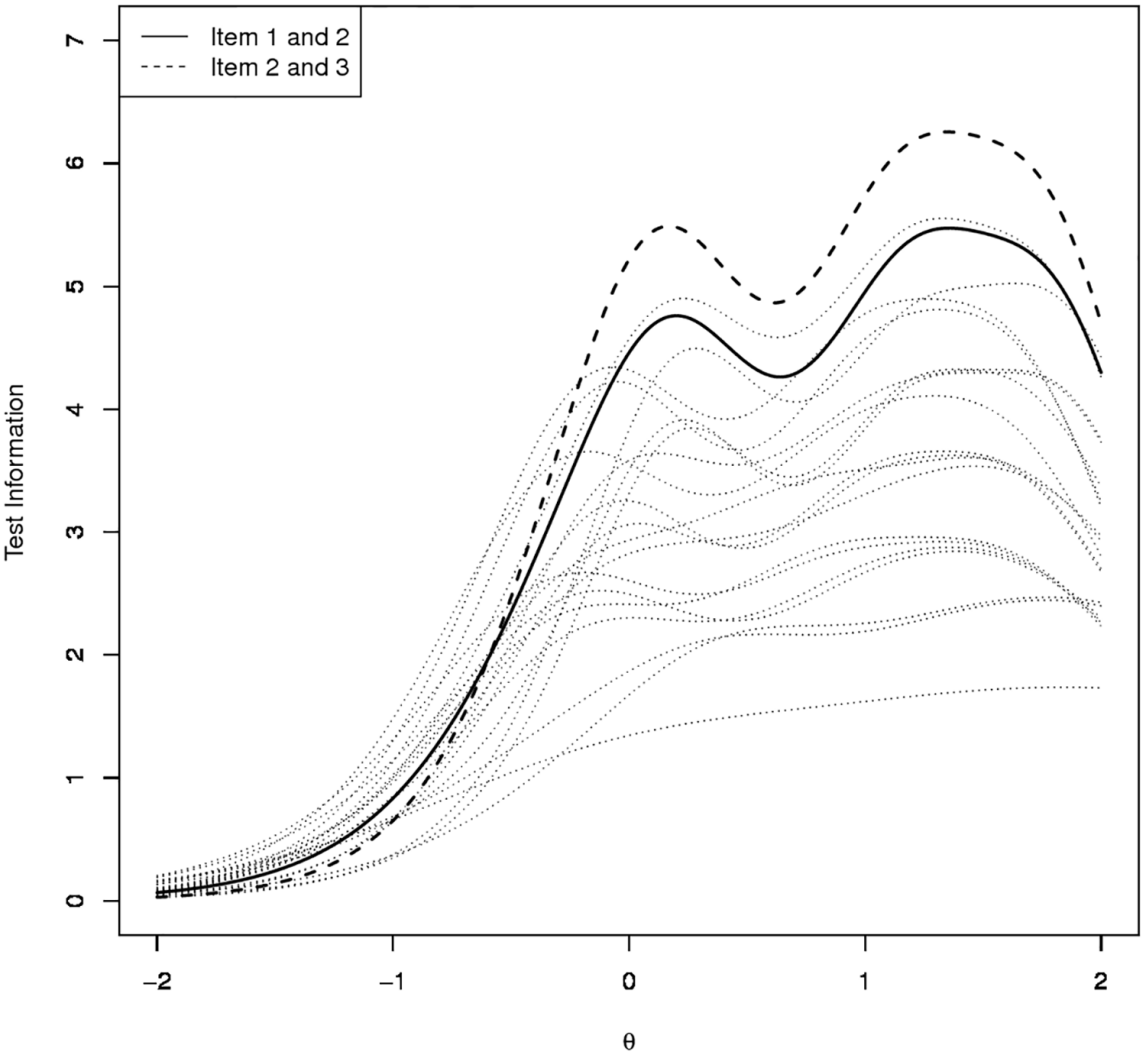
Test information curves for all two-item versions of the GAD-7.

As an example, consider the problem of constructing a crude two-item screening instrument. Then, the commonly applied choice of using the first and the second item [32,33] shows favorable properties compared to most of the other item pairs (see Fig 2). However, as Fig 2 depicts, an alternative (less favorable for discerning low to moderate anxiety but more favorable when the emphasis is on discriminating high values of anxiety) choice would be given by using item 2 (“Not being able to stop worrying”) and item 3 (“Worrying too much about different things”).

Finally, a further practical implication is provided by the possibility to derive a best predictor of a patient’s latent anxiety given his response pattern on the test (or a specifically chosen subtest). This aspect is discussed in the appendix where we provide R-Code to implement the inference.

## 4. Discussion

The present study showed that the GAD-7 scale does not violate basic requirements for a unidimensional scale. In contrast to the CFA approach, this hypothesis was examined with a bare minimum of additional modeling assumptions. The analysis of the scale within a graded response model yielded parameter estimates enabling best prediction of GAD (see the appendix) and the construction of ultra-brief measures for GAD. We emphasize that the best predictor is not automatically in line with the sum score (as already can be anticipated from the variation in item discrimination presented in Table 3). For example, using the response pattern x = (1,0,0,0,3,2,2) with sum score 8 and the pattern y = (1,0,1,0,1,2,2) with sum score 7 a discrepancy arises as the best predictor of the underlying latent anxiety is .16 vs .36 (the difference equals 20% of the standard deviation of the latent variable). Thus, in general the usage of the sum score should not be advocated unequivocally. In the above case, a one-point increase in an item (“Worrying too much about different things”) reflecting one of the two core DSM-5 criteria outweighs a two-point decrease in a comparably low discriminating item (“Hard to sit still”). In general, we advocate the usage of the best predictor (see supplement for the computation) instead of the sum score. Note also that unequal weightings of the items is also supported by the CTT analysis outlined at the end of section 3.1, wherein the composite score with maximal reliability shows remarkable discrepancies with regard. to uniform weighting. Of course, such a statement is based on test theory (on the interdependencies of the items) and subject to the limitation that no objective external measure of generalized anxiety was available to “back-up” this result via regression analysis. As the item information curves (Fig 1) clearly show, items differ substantially in their discriminating power. Thus assigning equal weights–which is commonly done in practice—seems counterproductive. In addition, item information curves suggest that the uncertainty of a particular point estimate is not uniform across the latent continuum. More specifically, confidence intervals are smaller for high values of anxiety than for low values of anxiety (Fig 1).

The topic of constructing ultra-brief assessment tools based on the GAD-7 items was already implicitly tackled by the computation of the test information curves for all item pairs–depicted in Fig 2. Our results provide additional evidence for the usage of the first item pair (“Feeling nervous anxiety or on edge”,”Not being able to stop worrying”)–as was suggested in Kroenke et al. [13] on the basis of receiver-operating characteristics. This item pair compares favorably to nearly all possible alternatives. The only pair which could be superior is given by the second (“Not being able to stop worrying”) and third item (“Worrying too much about different things”). However, item content analysis suggests that this pair reflects closely related aspects of GAD. Therefore evidence from three distinctive viewpoints (ROC; IRT and item content) suggests the first item pair as a reasonable ultra-brief assessment tool.

Thus, the main findings which are tied to the practical and clinical application are as follows:

- The GAD-7 provides a psychometrically sound instrument which can be incorporated as an economic and easily applicable instrument in primary care practices to single out patients with anxiety disorders with the ultimate aim to apply proper treatments and to reduce economic costs in the long-term.
- The GAD-7 does not discriminate properly in the lower spectrum of anxiety. Therefore, its usage should be restricted to the detection of anxiety disorders.
- If time pressure in daily primary care routine demands, then the practitioner can resort to a ultra brief measure consisting of only the first two items. It should however, be borne in mind, that the standard error of the estimate of any measure which consists of only two items is quite high, so that the ultra-brief version should only be used with great caution. In general, one should only cut corners and use these ultra-brief versions when time limits really dictate that the application of the whole scale is infeasible.We conveyed the message that the application of a simple unweighted sumscore is not suitable and offered a method to score the test items in a statistically optimal way (see appendix). Yet, we realize that practical constraints dictate a faster and more easily applicable scoring method. In order to obtain a quantitative scoring, we can substitute for the simple score a score with weights which are given in section 3.2 or we can use (as a crude approximation) the loadings given in Table 3. In fact, we expect that any method which keeps the maximum weight discrepancy at 3 and which incorporates the notion that the first four items are superior to the last three items will be more suitable than the usage of the ordinary sumscore.

Especially this last observation is crucial. To demonstrate the impact of weighting items properly (and thus of not treating them interchangeably), note that the response pattern (2,2,1,0,0,0,0) leads to the almost the same estimate of latent anxiety as the response pattern (0,1,0,0,3,3,3). However, using the ordinary classification based on the simple sumscore the first pattern would be classified as low levels of anxiety (sum score 5), whereas the second pattern would be characterized as exhibiting moderate levels of anxiety. We can also show that–no matter how severe the last four items are scored, if the first three are only at moderate to low levels, then very large estimates of anxiety are not possible. For instance, the extreme response pattern (1,1,1,3,3,3,3), which would ordinarily be classified “severe” (sumscore 15) leads to an estimate of 1.3 which is high–but still 10% of patients in primary care would show higher levels of anxiety.

As potential limitations of this study, no other measure of generalized anxiety was applied to validate the scale and its weighted versions. Thus, the conclusions of this study are based on purely test theoretical analyses. In addition, it has always to be assumed that the patients who participated in the study are not selectively biased in terms of item associations in comparison to the patients who denied to participate in the study. Finally, as items were always presented in the same order, the potential dependency of the response patterns on the particular sequence of item presentation cannot be analyzed. Although this type of assessment is rarely done in practice and research, this does not imply that the underlying phenomenon is not a threat to the validity of the scale.

Finally, we now head towards a short comparison of our results with one of the rare studies employing the IRT paradigm to the GAD-7 –the study of Schalet et al. [8]. The authors studied the properties of the GAD-7 (simultaneously with other measures of anxiety) within a large sample recruited via Internet survey providers from the general US population. Although our underlying sampling population differs, the results regarding the item discrimination parameters are comparable–that is the ratio of two different item discrimination parameters is similar in both studies (note that only ratios should be interpreted as ratios are invariant towards a shift in the scale of the latent variable). The most dissimilar ratios (e.g. 1.59 in our study vs 1.12 in Schalet et al.–both referring to the ratio between the fourth and the fifth item) all involve comparisons with the fifth item (“being so restless that it is hard to sit still”). Thus the impact of the fifth item is downgraded in our sample relative to the sample of Schalet et al. Note that Schalet et al. use an extended item battery (with the GAD-7 as some part of it) to estimate the item parameters. Hence, some differences are to be expected due to the potential influence of the inclusion of other items–not related to the GAD-7. Additionally, due to computational stability, we use logistic errors instead of normal errors. With this slight discrepancy in mind, the measurement attributes of the items in these distinct samples can be treated as comparable.

Nevertheless, we must emphasize, that the underlying model (graded response model) does show some misfit and that–based on the pattern of posterior predictive p-values–an improved model could be formulated by including an additional item parameter which captures local dependencies between consecutive items.

## Supporting information

S1 Fig Category response functions of the GAD-7-items. (TIF)

S2 Fig Test information curve of the complete GAD-7. (TIF)

S1 Appendix R-Code for the computation of the best predictor. (DOCX)

S1 Dataset Dataset of the study. (SAV)
