# Supplementary figures and images for: Psychometric analysis of the Generalized Anxiety Disorder scale (GAD-7) in primary care using modern item response theory
Source: PLoS One. 2017 Aug 3;12(8):e0182162. doi: 10.1371/journal.pone.0182162 (PMC5542568; doi:10.1371/journal.pone.0182162)

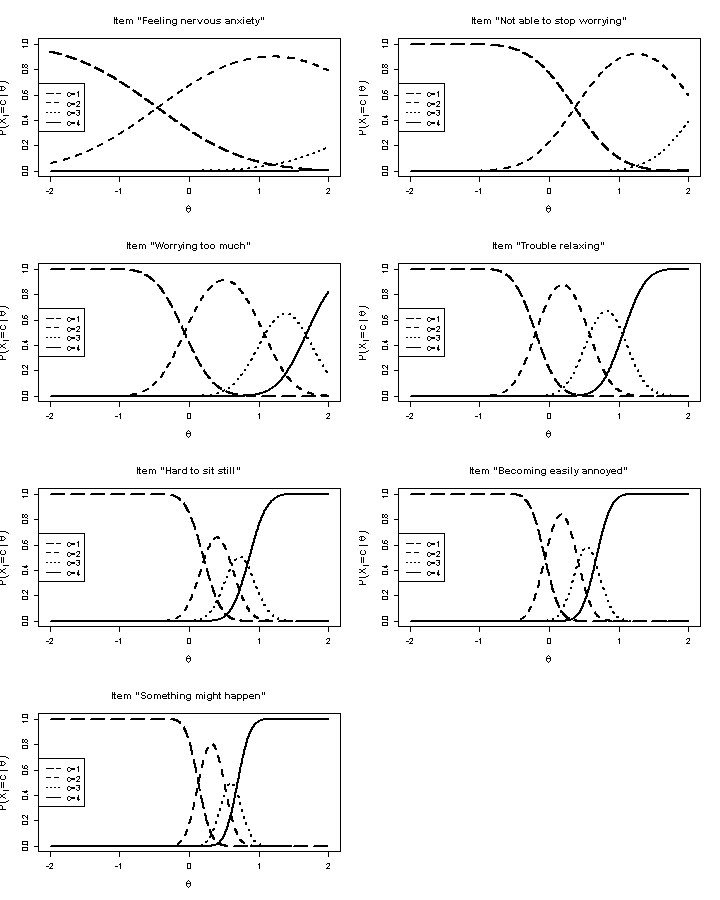

Supplement: S1 Fig — (TIF) [file pone.0182162.s001.tif]

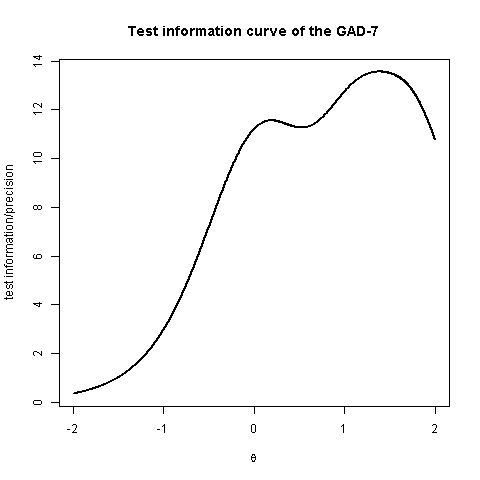

Supplement: S2 Fig — (TIF) [file pone.0182162.s002.tif]
