# Supplementary material for: Psychometric analysis of the Generalized Anxiety Disorder scale (GAD-7) in primary care using modern item response theory
Source: PLoS One. 2017 Aug 3;12(8):e0182162. doi: 10.1371/journal.pone.0182162 (PMC5542568; doi:10.1371/journal.pone.0182162)
Supplement: S1 Appendix — (DOCX) [file pone.0182162.s003.docx]

**Appendix**

This appendix provides R-Code for the computation of the best predictor (see chapter 7 of (34)) of generalized anxiety given the response pattern of a patient. This way of predicting GAD is statistically more efficient than simple sum score computation. For the numerical derivation of the best predictor (the expected a-posterior estimator) we use the fundamental identity of importance sampling (see ch. 3 in (35)).

For the handling of missing data we propose the following procedure: If data are missing at random (in particular, if they are missing by “design”) then compute the EAP using the given function with the observed response pattern (and the corresponding item parameters). If on the other hand, it can not safely be assumed that responses are missing at random, then perform a sensitivity analysis by calculating the EAP for a range of possible response patterns – i.e. compute various EAPs based on the observed response pattern augmented with additional possible values of the missing responses.

*R-Code for the computation of the best prediction of the GAD-score*

#This function computes the (conditional) probability of response pattern x [note that the #coding for each item is 1-4 instead of 0-3!] for a test taker with latent ability theta. [a denotes #the vector of item discrimination and tau the matrix of item thresholds]

grm_cond <- function(theta,a,tau,x)

{p <- length(a)

tau <- rbind(rep(-Inf,p),tau,rep(Inf,p))

z <- plogis(diag(tau[x+1,])-a*theta)-plogis(diag(tau[x,])-a*theta)

joint <- prod(z)

return(joint)

}

#vgrm is a vectorized form of the above function - allowing as an input not only a single #latent ability but a whole vector of latent abilities (this function is just a tool for the #computation and need not be adjusted)

vgrm <- Vectorize(grm_cond,"theta")

#The main function!

#Computes the EAP estimate of a test takers latent ability via use of the fundamental identity #of importance sampling.

#Inputs: a: vector of item discrimination; tau: the matrix of item thresholds; x: the observed #response pattern of the test taker.

#Output: Estimate for the test takers latent ability (GAD-score) based on the theory of "best #prediction"

sample_nor <- function(a,tau,x)

{theta <- rnorm(100000,0,1)

cond <- vgrm(theta,a=a,tau=tau,x=x)

return(mean(theta*cond)/mean(cond))

}

#Demonstration of the function via the GAD7:

#The item parameters of the GAD7

a <- c(2.6,3.5,3.1,2.7,1.7,1.7,2.3)

tau <-matrix(c(‑0.5,2.8,4.3,0.7,4.2,6.4,‑0.2,3.2,5.1,‑0.8,2.3,

4.3,1,2.9,4.3,‑0.4,2.4,4.1,0.9,3.5,4.9),nrow=3)

#Three possible response patterns

x=c(2,1,1,1,4,3,3)

x1=c(2,2,1,1,4,3,3)

x2=c(2,3,1,1,4,3,3)

#Three estimates corresponding to the chosen response pattern

sample_nor(a,tau,x)

sample_nor(a,tau,x1)

sample_nor(a,tau,x2)

**References**

**34.** Searle SR, Casella G, McCulloch CE. Variance components: John Wiley & Sons; 2009.

**35.** Robert C, Casella G. Monte Carlo statistical methods: Springer Science & Business Media; 2013.
